# Biocompatibility of Four Common Orthopedic Biomaterials Following a High-Salt Diet: An In Vivo Study

**DOI:** 10.3390/ijms18071489

**Published:** 2017-07-11

**Authors:** Mathieu Lecocq, Cécile Bernard, Marie Solenne Felix, Jean-Marc Linares, Julien Chaves-Jacob, Patrick Decherchi, Erick Dousset

**Affiliations:** 1 Aix-Marseille Université, CNRS, Institut des Sciences du Mouvement: Etienne-Jules MAREY (UMR 7287), Equipe Plasticité des Systèmes Nerveux et Musculaire (PSNM), Faculté des Sciences du Sport, CC910, 163, Avenue de Luminy, 13288 Marseille CEDEX 09, France; mathieu.lecocq@univ-amu.fr (M.L.); cecile.bernard@gmx.fr (C.B.); marie-solenne.felix@univ-amu.fr (M.S.F.); patrick.decherchi@univ-amu.fr (P.D.); 2 Aix-Marseille Université, CNRS, Institut des Sciences du Mouvement: Etienne-Jules MAREY (UMR 7287), Equipe Conception Bio-Inspirée (CBI), IUT d’Aix-en-Provence 413, avenue Gaston Berger, 13625 Aix-en-Provence CEDEX, France; jean-marc.linares@univ-amu.fr (J.-M.L.); julien-Chaves-jacob@univ-amu.fr (J.C.-J.)

**Keywords:** polyetheretherketone, aluminum oxide, chrome-cobalt, titanium alloy, cell viability, implant-to-bone adhesion

## Abstract

Nowadays, salt consumption appears to be drastically above the recommended level in industrialized countries. The health consequences of this overconsumption are heavy since high-salt intake induces cardiovascular disease, kidney dysfunction, and stroke. Moreover, harmful interaction may also occur with orthopaedic devices because overconsumption of salt reinforces the corrosive aspect of biological tissues and favors bone resorption process. In the present study, we aimed to assess the in vivo effect of three weeks of a high-salt diet, associated (or not) with two weeks of the neuro-myoelectrostimulation (NMES) rehabilitation program on the biocompatibility of four biomaterials used in the manufacture of arthroplasty implants. Thus, two non-metallic (PEEK and Al_2_O_3_) and two metallic (Ti6Al4V and CrCo) compounds were implanted in the rat tibial crest, and the implant-to-bone adhesion and cell viability of two surrounded muscles, the *Flexor Digitorum* (FD) and *Tibialis Anterior* (TA), were assessed at the end of the experiment. Results indicated lower adhesion strength for the PEEK implant compared to other biomaterials. An effect of NMES and a high-salt diet was only identified for Al_2_O_3_ and Ti6Al4V implants, respectively. Moreover, compared to a normal diet, a high-salt diet induced a higher number of dead cells on both muscles for all biomaterials, which was further increased for PEEK, Al_2_O_3_, and CrCo materials with NMES application. Finally, except for Ti6Al4V, NMES induced a higher number of dead cells in the directly stimulated muscle (FD) compared to the indirectly stimulated one (TA). This in vivo experiment highlights the potential harmful effect of a high-salt diet for people who have undergone arthroplasty, and a rehabilitation program based on NMES.

## 1. Introduction

According to the World Health Organization [1], food consumption in industrialized countries is frequently described as rich in salt because sodium (Na^+^) concentration drastically exceeds the recommended level (1.5 g/day and no more than 2.3 g/day). Its intake averages 150 mmol/day (3.5 g/day), a figure far in excess of the minimal daily requirement (0.18 g/day or 8 mmol/day) to replace losses under conditions of maximal adaptation and without sweating [2]. Two factors exacerbate this trend: (1) Ignorance of risk associated with a high-salt diet; and (2) The inability to know the amount of Na^+^ in industrial food leading to an underestimation of its amount intake [3,4]. For example, it is reported that 75% of the daily sodium intake of the US population comes from salt added by food manufacturers and restaurants, making it extremely difficult for consumers to follow a low-sodium diet [3,5]. This overconsumption of Na^+^ leads to a high prevalence of cardiovascular disease, kidney dysfunction, and stroke [6,7]. Furthermore, high-salt intake promotes metabolic acidosis [8,9,10] which impairs bone metabolism leading to bone degradation and osteoporosis [11,12,13,14,15]. Finally, aging is associated with a down-regulation of acid-base balance [6,16] leading to a reinforcement of the harmful effect of the salt on bones and to an increase in normal age-related process of osteoporosis.

Thus, the adverse effect of a high-salt diet on bones degradation and the high prevalence of arthroplasty prescribed every year for elderly people [17] should be taken into consideration as a potential risk factor of implant loosening. Indeed, osteolysis, which is amplified by excessive salt consumption, is recognized as a major factor of such a loosening process [18,19,20,21,22]. Moreover, in addition to the direct effect on bone resorption, Na^+^ and also Cl^−^ could amplify the corrosive aspect of surrounding tissues on the implant surface leading to the production of wear debris promoting bone resorption [23,24,25,26]. Salt overconsumption leads to a richer electrolytic environment that changes the electrochemical balance of biological tissues, thus increasing the chemical stress on biomaterial surfaces and a higher wear debris dissemination [27,28,29]. Furthermore, this harmful phenomenon could be amplified by large currents generated by the excess of ion and electron flow occurring during electrochemical reactions between the corroding metallic surface and the electrolytes [30]. It might be possible that electrical stimulation used after arthroplasty surgery for physical rehabilitation could amplify the propagation of electrical currents. Thus, an uncontrolled risk of electric current conductions through biological tissues could be dangerous for prosthesis integrity and cell viability in the nearby tissues. In a previous study, we showed a decrease of muscle cell viability after two weeks of neuro-myoelectrostimulation (NMES) in rats submitted to a high-salt diet. Moreover, in the same study, we reported that number of dead cells was higher when a Ti6Al4V biomaterial was implanted near the stimulation area [31]. Although this is worrying for patients who resort to arthroplasty, further studies are needed to confirm these results, in particular, to what extent such a deleterious effect could occur with other commercialized biomaterials.

Thus, we aimed to compare interaction between a high-salt diet and four orthopedic biomaterials widely used in the manufacture of arthroplasty implants (metallic: Ti6Al4V and CrCo and non-metallic: PEEK and Al_2_O_3_) on animals submitted (or not) to a NMES program. We hypothesized that a high-salt diet could result in a higher number of dead cells near the implant and weaken implant anchorage for the metallic biomaterials compared to the non-metallic ones, and that the addition of an electrical current could amplify this trend.

## 2. Results

### 2.1. Implant-To-Bone Adhesion

**Figure 1:**
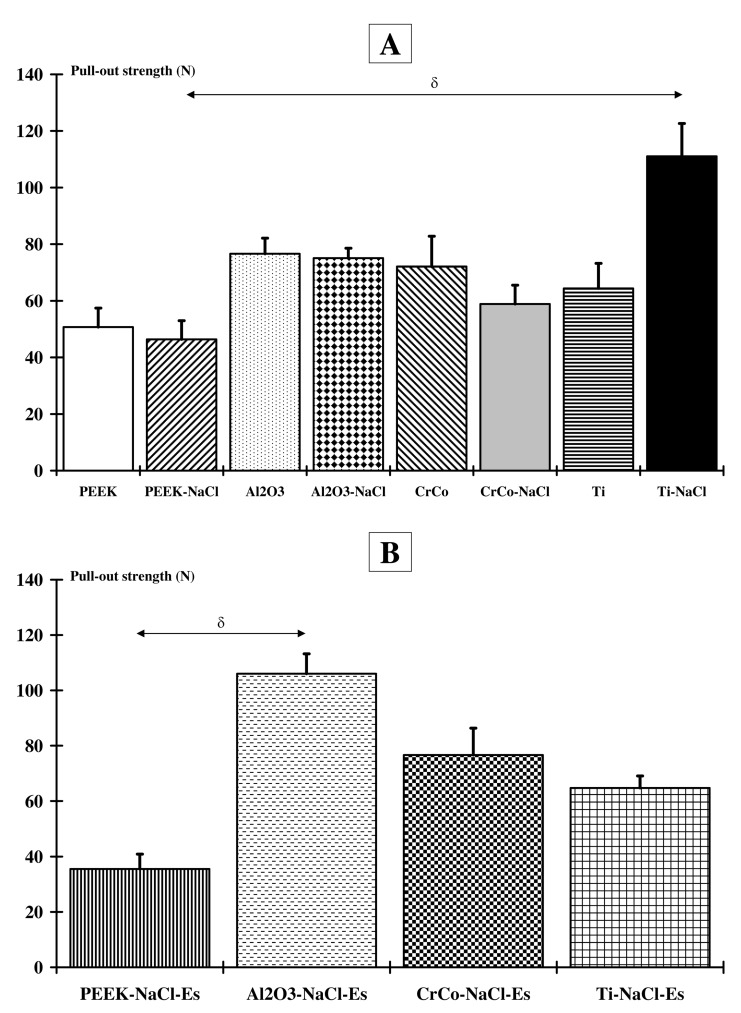
Implant-to-bone adhesion. (**A**) Comparison of the maximum load value for all implanted animals with and without a high-salt diet; (**B**) Comparison of the maximum load value for all implanted and neuro-myoelectrostimulated animals that received a high-salt diet. A significant difference is found between the PEEK-NaCl and Ti-NaCl groups (δ: *p* < 0.05), and between the PEEK-NaCl-Es and Al_2_O_3_-NaCl-ES groups (δ: *p* < 0.05).

Without NMES application (Figure 1A), implant-to-bone adhesion was significantly higher for the Ti-NaCl group (111.03 ± 11.61 N, *p* < 0.05) compared to the PEEK-NaCl group (46.38 ± 6.63 N). After NMES application (Figure 1B), pull-out force was significantly lower (*p* < 0.05) for PEEK-NaCl-Es (35.53 ± 5.34 N) compared to the Al_2_O_3_-NaCl-Es group (106.01 ± 7.22 N). No additional significant differences were found between all the other groups.

**Figure 2:**
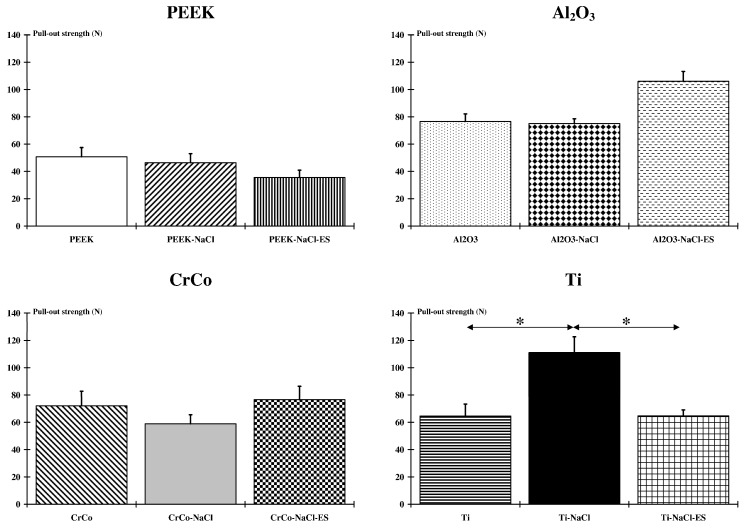
Biomaterial and bone adhesion. Comparison of implant-to-bone adhesion for each biomaterial does not indicate a difference between each condition for the PEEK, Al_2_O_3_, and CrCo groups. Only animals receiving the titanium alloy show a significant (*: *p* < 005) difference between the Ti-NaCl group and the other two groups (Ti and Ti-NaCl-Es).

Considering each biomaterial independently, comparison of implant-to-bone adhesion did not indicate a difference between each condition for the PEEK, Al_2_O_3_, and CrCo groups. Only animals receiving the titanium alloy showed a significant (*p* < 005) difference between the Ti-NaCl group and the other two groups (Ti and Ti-NaCl-Es) (Figure 2). On the other hand, for each group the normalized value (expressed with respect to non-treated groups) of the NaCl and NaCl-Es conditions were, respectively, 84.4% and 74.4% for PEEK, 101% and 141.34% for Al_2_O_3_, 89.35% and 126.9% for CrCo, and 186.5% and 110% for Ti.

### 2.2. Muscle Damage

**Figure 3:**
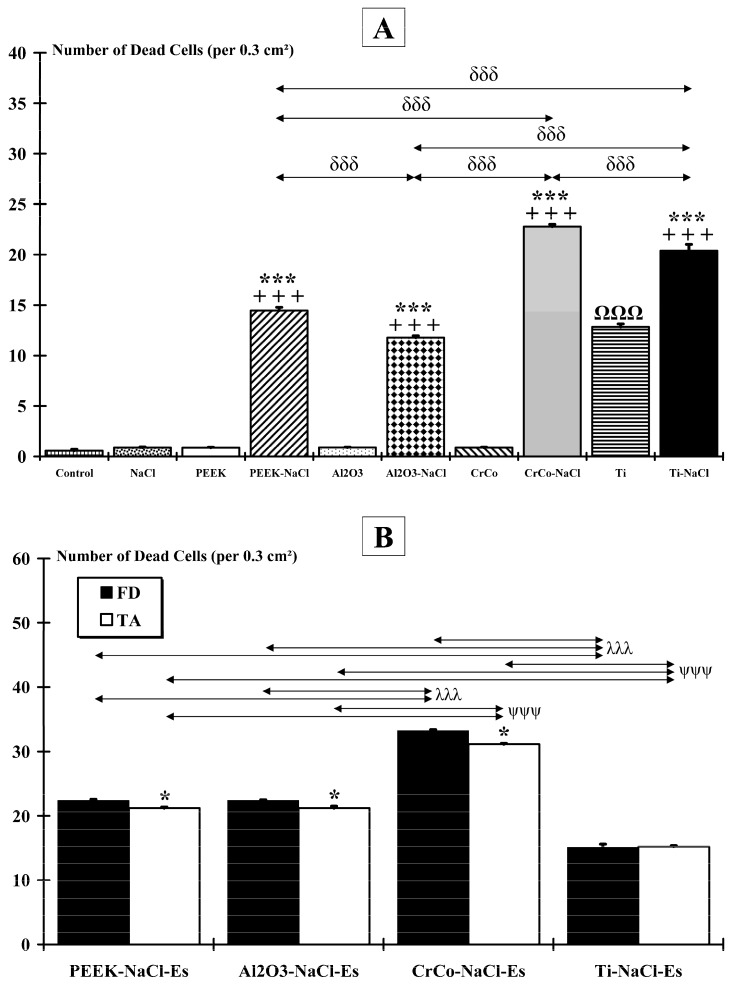
Muscle damages. Antibodies directed against activated caspase-3 are used on *flexor digitorum* (FD) muscle to evaluate the number of dead cells per surface unit. (**A**) Except for the Ti group (ΩΩΩ: *p* < 0.001), the implant alone does not induce muscle damage compared to non-implanted animals (Control and NaCl). However, when implanted animals are submitted to a very salty diet, a significant (***: *p* < 0.001) number of dead cells are observed compared to non-implanted animals. Finally, all implanted animals exhibit a significant (+++: *p* < 0.001) increase in the number of dead cells in a high-salt diet condition compared to a normal diet. The δ symbol indicates intergroup differences (δδδ: *p* < 0.001); (**B**) Comparison between the two muscles indicates that the number of dead cells is significantly (*: *p* < 005) lower in the indirectly stimulated muscle (tibialis anterior, TA) compared to the directly stimulated one (FD). The λ symbol indicates FD intergroup differences (λλλ: *p* < 0.001). The ψ symbol indicates TA intergroup differences (ψψψ: *p* < 0.001).

Without NMES application and a high-salt diet (Figure 3A), for FD muscle, no significant number of dead cells was observed in Control (0.56 ± 0.14) and NaCl (0.87 ± 0.07) groups, and no difference was found between these two groups. Except for the Ti group (12.83 ± 0.31; *p* < 0.001), no significant difference was found between only implanted groups (PEEK (0.86 ± 0.06), Al_2_O_3_ (0.88 ± 0.05) and CrCo (0.87 ± 0.06)) and non-implanted (Control and NaCl) groups. Furthermore, compared to non-implanted animals, a high-salt diet induced a significant number of dead cells in all implanted groups (whatever the biomaterial): PEEK-NaCl (15.45 ± 0.33; *p* < 0.001), Al_2_O_3_-NaCl (11.78 ± 0.17; *p* < 0.001), CrCo-NaCl (22.78 ± 0.21; *p* < 0.001), and Ti-NaCl (20.39 ± 0.62; *p* < 0.001). Finally, for each biomaterial, a significant (*p* < 0.001) higher number of dead cells was observed when salt supplementation was given compared to the condition without a high-salt.

With NMES application and with a high-salt diet (Figure 3B), statistical analysis revealed that the number of dead cells in the directly stimulated muscle (FD) was significantly (*p* < 0.001) lower for Ti-NaCl-Es (15.08 ± 0.54) compared to all the other groups: PEEK-NaCl-Es (22.37 ± 0.17), Al_2_O_3_-NaCl-Es (22.37 ± 0.10), and CrCo-NaCl-Es (33.22 ± 0.17). The latter showed the highest number of dead cells, which significantly (*p* < 0.001) exceeded that of other groups. No difference was found between the PEEK-NaCl-Es and Al_2_O_3_-NaCl-Es groups. Finally, except for the Ti-NaCl-Es group, the number of dead cells in the indirectly stimulated muscle (TA) appeared to be significantly lower than those of the FD muscle: PEEK-NaCl-Es (21.2 ± 0.17; *p* < 0.05), Al_2_O_3_-NaCl-Es (21.2 ± 0.28; *p* < 0.05), and CrCo-NaCl-Es (31.11 ± 0.17; *p* < 0.05).

**Figure 4:**
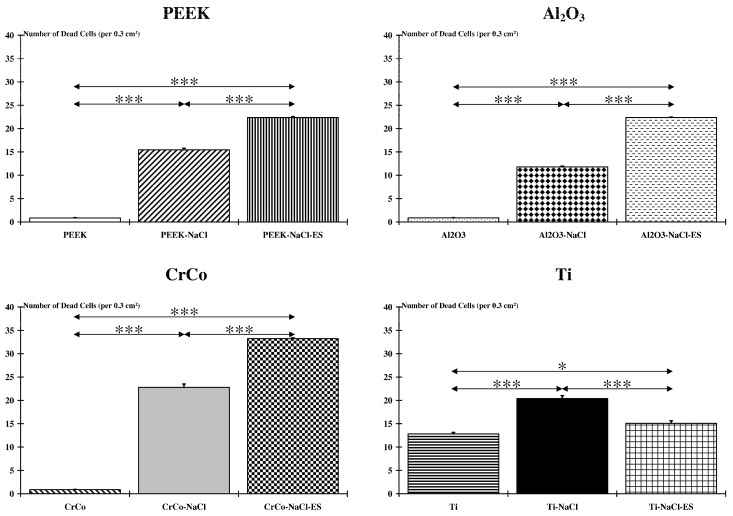
Biomaterial and muscle damages. Except for animals receiving titanium alloy, NMES combined to a high-salt diet constitutes the most deleterious condition for tissues surrounding the implant. However, whatever the biomaterial considered, a high-salt diet and NMES increase the number of dead cells compared to implanted groups that do not follow the NMES program, or to groups submitted to a normal diet (*: *p* < 0.05; ***: *p* < 0.001).

Considering each biomaterial independently, except for animals receiving titanium alloy for which the number of dead cells was highest in the Ti-NaCl group, NMES combined with a high-salt diet constituted the most deleterious condition for tissues surrounding the implant. Indeed, the number of dead cells was significantly higher (*p* < 0.001) in the PEEK-NaCl-ES, Al_2_O_3_-NaCl-Es, and CrCo-NaCl-Es groups compared to the PEEK-NaCl, Al_2_O_3_-NaCl, and CrCo-NaCl groups or the PEEK, Al_2_O_3_ and CrCo groups, respectively. However, whatever the biomaterial considered, a high-salt diet and NMES increased the number of dead cells compared to implanted groups that did not follow the NMES program, or compared to implanted groups submitted to a normal diet (Figure 4). For each group, a normalized value (expressed with respect to non-treated groups) of the NaCl and NaCl-Es conditions were, respectively, 1815% and 2631% for PEEK, 1362% and 2582% for Al_2_O_3_, 2598% and 3907% for CrCo, and 163.4% and 118.7% for Ti.

## 3. Discussion

Nowadays, salt consumption of industrialized countries appears to be drastically above the recommended level [1,2]. Beyond the well-known negative effect on the cardiovascular system, excess salt consumption could have serious consequences on bone metabolism, particularly for elderly people who are often subject to kidney dysfunction and osteoporosis [5,6,14,15,16]. Indeed, low-grade metabolic acidosis mediated by the excess of salt consumption directly induces the dissolution of bone mineral by both stimulating osteoclast-mediated bone resorption and inhibiting osteoblast-mediated bone formation [9,32,33]. It was showed that a small decrease of tissue pH following a high-salt diet could be responsible for important bone resorption [34]. Thus, in an orthopedic context, high-salt consumption could favor osteolysis, an important factor of prosthesis loosening [18,19,20,22]. Moreover, it appears that a pH decrease reinforces the corrosive aspect of biological tissues, and a high ionic content could promote large current propagation from a metallic implant, which may potentially impair biological tissues [27,28,30]. In the present study, the influence of three weeks of a high-salt diet on the bone-adhesion of four common biomaterials implanted in the rat tibial crest and the cell viability of two surrounding muscles (FD and TA) were investigated. Moreover, for each biomaterial, one additional group performed two weeks of an NMES session, based on a human program, during the period of salt supplementation. Although this study does not allow us to identify the systematic effect of a high-salt diet on implant adhesion, it seems that interaction between high-salt intake and metallic (Ti6Al4V and CrCo) or non-metallic (PEEK and Al_2_O_3_) implants have an adverse effect on surrounding muscle cell viability.

### 3.1. Implant-To-Bone Adhesion

Interestingly, whatever the condition, PEEK adhesion strength tended to be worse compared to other biomaterials. More specifically, in a high-salt diet condition, PEEK was subject to low adhesion strength compared to the Ti biomaterial. Furthermore, in a high-salt diet condition associated with the NMES program, PEEK adhesion strength was lower than that of Al_2_O_3_. In the literature, PEEK is usually described as an efficient alternative to the systematic use of metallic alloys in the orthopedic process as it presents suitable chemical resistance and low cytotoxicity, and because it has mechanical properties similar to those of human bones [35,36,37]. Furthermore, it was demonstrated that PEEK didn’t generate an adverse effect on osteoblast and fibroblast activity in cell culture [38]. Morrison and co-workers suggested that PEEK could stimulate osteoblast protein content and encourage bone cell proliferation around material [39]. Paradoxically, more recent studies based on clinical reports and animal experimentations emphasized doubts about the capacity of PEEK biomaterials to integrate the surrounding bone tissue well [35,40,41]. Our results support, as was also recently observed in condition of a normal diet associated with NMES [42], that PEEK osseointegration is limited compared to other biomaterials in the condition of a high-salt diet associated (or not) with NMES, although no difference was found in the condition of a normal diet and without NMES.

Other studies also showed limited Al_2_O_3_ direct bonding with bone cells [43,44,45]. However, in our study, whatever the condition, no difference was found in adhesion strength compared to Ti and CrCo, which are renowned to have good osseointegration [46,47]. Only an effect of NMES and a high-salt diet was identified for Al_2_O_3_ and Ti6Al4V implants, respectively. Paradoxically, the implant-to-bone adhesion revealed that the strength required for implant loosening was very high for the Ti-NaCl group. This result is in disagreement with many studies that demonstrate the harmful effects of a high-salt diet and an increase of bone resorption [11,12,48]. Indeed, it was reported that a high-salt diet provokes an increase of tissue acidity leading to bone loss [9]. The latter might be explained by an increase in osteoclast activity in response to the degradation process [49]. Thus, we could hypothesize that this increase in osteoclastic activity leads to an increase in osteoblastic activity, which in turn amplifies adhesion of the Ti implant to bone. Moreover, the addition of NEMS could result in an increase in blood flow [50,51] thereby reducing accumulation of ions around the implant and finally reducing this hypothetical compensatory reaction, explaining the decrease in implant-to-bone adhesion.

### 3.2. Number of Dead Cells

In the present study, contrary to titanium alloy, we observe that polyetheretherketone, aluminum oxide and chrome-cobalt implants had no effect on cell viability when they were not associated to high-salt diet. The absence of cell death in the PEEK, CrCo, and Al_2_O_3_ groups and the presence of dead cells in the Ti group suggest that muscle damages were not due to surgical procedure but to the interaction of the Ti6Al4V implant with biological tissues. This adverse reaction could have originated from the corrosion process currently observed between metallic alloys and biological tissues [22,52,53,54]. However, in our study, considering the short term exposure of Ti6Al4V to biological environment, this hypothesis has to be taken with caution.

As previously described [31], a high-salt diet *per se* has no effect on muscle cell viability in the non-implanted animals. However, a high-salt diet induced a deleterious effect on muscle cell viability in animals receiving a titanium implant [31]. Here, we also observe a deleterious effect on the surrounding tissue when PEEK, CrCo, and Al_2_O_3_ were associated with a high-salt diet. The literature reported that high saline solutions exerted adverse effect on the metallic surface by promoting corrosion [24,28]. Thus, in vivo, the main hypothesis is that intake of a high amount of salt lowers the local pH of the implant environment, leading to the reinforcement of the corrosive aspect of biological tissues on the CrCo and Ti6Al4V surfaces and to wear debris dissemination that, in turn, promotes cell necrosis [55]. Moreover, it was demonstrated that in patients bedridden for two weeks a high-salt diet accelerated bone resorption and muscle protein loss [9,48]. Finally, in the present study, we also report that a high-salt diet exerted an adverse effect on the non-metallic compound. To the best of our knowledge, no previous study reported such harmful interaction with non-metallic implants. However, we could hypothesize that because of the permittivity of the inert biomaterials such as PEEK and Al_2_O_3_ [56], the insertion of a dielectric component into an electrical field created by the NMES may induce a local electrical phenomenon on the surface of the implant. This phenomenon, which can be assimilated with an electrical phenomenon in a capacitor, could contribute to an increase in the number of dead cells in the context of a high-salt diet. Thus, whatever the mechanism causing cell death, a high-salt diet has deleterious effects on the biological environment of a metallic and non-metallic implant.

In the present study, we observe that the deleterious effects of a high-salt diet on the surrounding tissue were increased when animals with PEEK, CrCo, and Al_2_O_3_ implants were submitted to a NMES program. The increase in the number of dead cells could result in an increase in Na^+^ and Cl^−^ ion content in tissue, leading to an increase in NMES-induced current potentiation and propagation through biological tissue because of a greater tissue conductance [30,57]. Furthermore, among biomaterials, CrCo seems to be more subject to harmful interaction between salt and electrical current because it presents the highest number of dead cells in both NaCl and NaCl-Es conditions. Even if this material is still considered biocompatible, clinical cases and scientific studies show that CrCo material induces hypersensitivity, marked immune response, metallosis, and detrimental effects on surrounding tissues [25,58,59,60,61,62,63]. Interaction with high Na^+^ and Cl^−^ ions content may exacerbate these negative effects as suggested by the results we obtained. Paradoxically, the amount of muscle damage observed in the Ti-NaCl-Es group was lower than that of the Ti-NaCl group. It is likely that the same process of degradation occurred in the two groups. However, as previously described, blood flow increases caused by chronic stimulation [50,51] may decrease ion accumulation and thus reduce the level of corrosion. Such a process might explain the lower cell death rate observed in the stimulated groups. Finally, except for the Ti-NaCl-Es group, a specific effect of the electrical current could be identified on the directly stimulated muscle (FD) that exhibited a higher number of dead cells than the indirectly stimulated muscle (TA), indicating that tissues near the source of the current are more vulnerable than the distant tissues. Indeed, the closer the tissues are to the source of current, the more the current loops are numerous and large, and when moving away from the source they are fewer, dissipated in the conductive elements, and smaller (owing to decremental or electrotonic propagation which attenuates with distance).

### 3.3. Consideration in Regard to Normalized Value

In the case of implant-to-bone adhesion, the normalized data does not change our interpretation already described in Section 3.1. However, in regard to the number of dead cells in the normalized data, Ti could be considered as the most suitable biomaterials because this value appeared to be largely lower than that of other groups. Additionally, CrCo appeared to be the less suitable material. This interpretation should be considered with caution because the Ti basal line is much higher than that of other groups, meaning that Ti implants alone are already deleterious for the surrounding tissue.

## 4. Materials and Methods

### 4.1. Models

Seventy male Sprague Dawley rats (weighting approximately 400 g) were used in this study (Centre d’Elevage Roger JANVIER^®^, Le Genest Saint Isle, France). They were housed in cages with a 12 h light/dark cycle at 22 °C and received food and water ad libitum. An acclimation period of one week allowed animals time to stabilize in the new environment before the beginning of the experimentation.

Rats were randomly assigned to 14 experimental groups: (1) The control (*n* = 5) group in which no treatment and surgery were performed; and (2) The NaCl (*n* = 5) group in which animals were submitted to a high-salt diet for 3 weeks (no surgery was performed), and 4 other groups named according to the materials implanted on tibial crest, i.e., PEEK (polyetheretherketone), Al_2_O_3_ (aluminum oxide), CrCo (chrome-cobalt), and Ti (titanium alloy: Ti6Al4V). Each of these four groups were divided into 3 subgroups according to the diet (normal or high-salt diet) given and the muscle condition (without or with NMES). Thus, rats were randomized into the following groups: (3) PEEK (*n* = 5), (4) PEEK-NaCl (*n* = 5), (5) PEEK-NaCl-Es (*n* = 5), (6) CrCo (*n* = 5), (7) CrCo-NaCl (*n* = 5), (8) CrCo-NaCl-Es (*n* = 5), (9) Al_2_O_3_ (*n* = 5), (10) Al_2_O_3_-NaCl (*n* = 5), (11) Al_2_O_3_-NaCl-Es (*n* = 5), (12) Ti (*n* = 5), (13) Ti-NaCl (*n* = 5), and (14) Ti-NaCl-Es (*n* = 5).

### 4.2. Ethical Approval

All animal handling was performed according to French animal care guidelines. Our protocols were approved by the Animal Care Committees of Aix-Marseille Université and Centre National de la Recherche Scientifique. All experimenters were listed in the authorized personnel section of the animal research protocol or added to a previously approved protocol (License A 13 01306). Moreover, this study was conducted in accordance to the Guide for Care and Use of Laboratory Animals (U.S. Department of Health and Human Services, National Institutes of Health), and the European Community’s council directive of 24 November 1986 (86/609/EEC). Throughout the protocol, no clinical sign of pain or unpleasant sensation (i.e., screech, prostration, hyperactivity, or anorexia) or paw-eating behavior was observed.

### 4.3. Implant Design

According to a previous study [64], we used an implant designed to fit a part of the rat tibial crest without risking surrounding muscles injury during all the protocol. The implant size (4 × 3 × 3.5 mm^3^) was chosen so that the classical surgery procedure (surgical pliers, without binocular microscope) could be used for implantation. The geometry of the designed implant was the same for all experiments.

**Figure 5:**
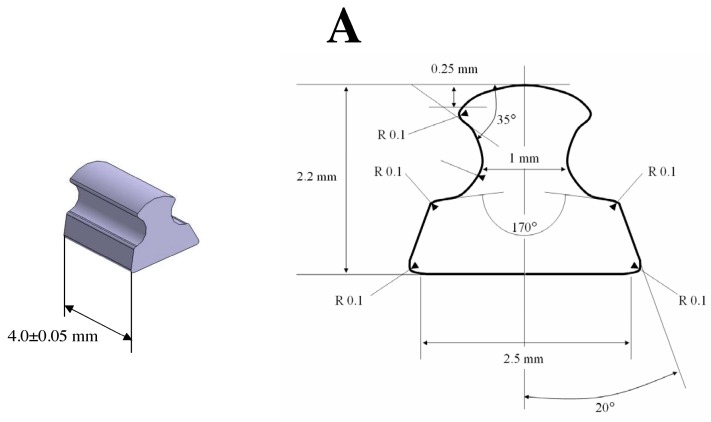

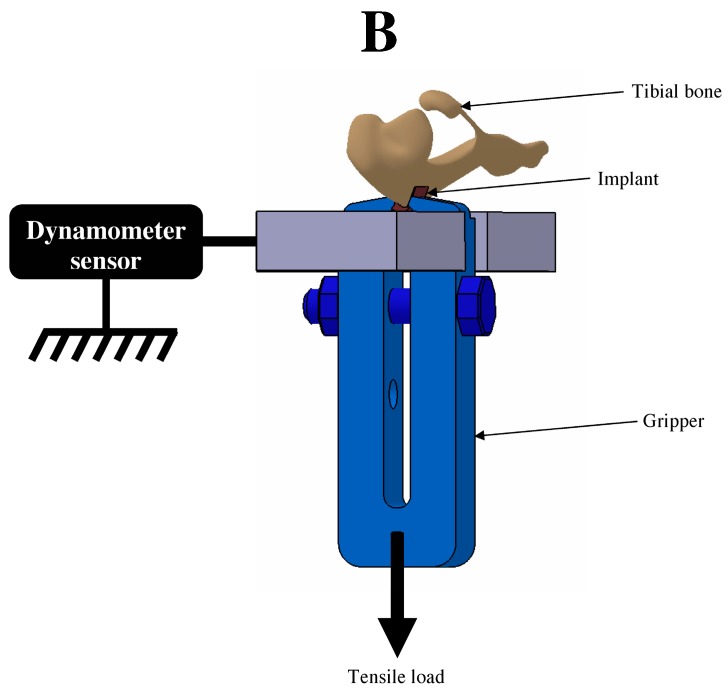
Implant characteristics and gripper design. (**A**) Shape, dimensions, and surface roughness of the implants. All implants respect this design. The surface roughness Ra is 0.8 μm; (**B**) A bolt is used to ensure the clamping of the gripper on the implant. Internal degrees of freedom of the gripper ensure the alignment of the implant during the tensile test.

Implants were machined from four different materials (metallic: Ti6Al4V and CrCo, and non-metallic: PEEK and Al_2_O_3_) by a 5-axes high-precision milling machine (US 20, Deckel Maho Gildemaster^®^, Leonberg, Germany). The computer-aided design and implant manufacturing were realized on CATIA^®^ V5 software (Dassault Systèmes^®^, Vélizy-Villacoublay, France). In order to avoid variability, all implants were identical and had a width of 3.5 mm, a length of 4 mm (largest base in contact with bone surface), and a height of 3 mm. A specific implant profile was realized to carry out the implant-to-bone adhesion test and the railway rail profile was used (Figure 5A). After machining, implants received only a cleaning treatment without a more particular surface treatment.

### 4.4. Surgical Protocol

All rats were anesthetized with an intraperitoneal injection of chloral hydrate (0.05 g/mL; 1 mL/100 g; (Sigma Life Science^®^, Saint-Louis, MO, USA)). The left hindpaw was shaved and a 1.5 cm skin incision was made along the tibial bone. Muscles in contact with the tibial bone were carefully separated from the bone in order to avoid muscle damage. A portion of the bone, similar to the volume of the implant, was removed from the tibial crest using a micro-milling/grinder machine (Dremel 300 series multitool, Bosch^®^, Mount Prospect, IL, USA). As in orthopedic surgery, the implant was anchored to the tibial bone with acrylic cement (CEMFIX 1 Teknimed S.A.S, L’Union, France). Cement was positioned on the surface of the implanted area. Then, the implant was fixed and cement was added on the bone-implant interface adhesion (this method allows free contact between a large part of the implant surface and the surrounding tissues). After a drying delay, muscles were sutured at two points on either side of the implanted area. Finally, the skin was sutured and animals received a local anesthetic (Lidocaine^®^ T7394c, Sigma-Aldrich^®^, Saint-Louis, MO, USA) subcutaneously around the implantation site to minimize pain.

### 4.5. High-Salt Diet

According to Safe^®^ Company (Safe S.A.S, Augy, France) which provided food for laboratory animals, we determined that the NaCl amount in the basic food ranged between 0.25% and 0.27% of food weight. Since most high-salt diets that were used in the scientific literature were composed with levels up to 8% NaCl [65,66], this value was chosen as a reference. In accordance with Oloyo et al., food pellets usually given to rats were mixed with water to form a smooth paste, then salt (7.75% of the total food weight) was added to reach the desired salt concentration of 8% and pellets were reconstituted before being dried for 24 h [67].

### 4.6. NMES Program

After surgical implantation, all animals were kept at rest during one week. Then, the PEEK-NaCl-Es, CrCo-NaCl-Es, Al_2_O_3_-NaCl-Es, and Ti-NaCl-Es groups performed NMES sessions five days a week for two weeks. An electrical currents feature was based on a common human clinical program employed for faster muscle reconditioning. In order to achieve stress-free sessions, rats were slightly anesthetized with a subcutaneous binary mixture injection of 0.65 mL of ketamine (Ketamine^®^ 1000, Virbac^®^, Carros, France) and 0.25 mL of chlorpromazine hydrochloride (Largactil^®^, 0.1 mL per 100 g, Aventis-Pharma^®^, Paris, France). Animals were placed in a supine position and immobilized by slightly tensed straps located on the thorax and abdomen. The ankle was firmly held with an angle of 90° to avoid movements and friction stimulations. Electrodes (ECG Electrodes universal^®^, Medical Chart Control^®^, Brie Comte Robert, France) were placed on the skin and positioned strictly above the flexor digitorum (FD) muscle according to the anatomical table. The correct electrodes position was insured by the fingers flexion induced by electrical stimulation. Moreover, because FD arises from the tibia below popliteus and from the head of the fibula, it was not in contact with the implant, thus avoiding any friction stress exerted on the implant.

The electrical stimulation program was composed of 40 stimulations at 75 Hz frequency and a 6.25 s duration separated by 20 s periods of active recovery (3 Hz stimulation). The choice of this program was based on literature indicating that stimulation with high frequencies applied to the quadriceps muscle after a total knee arthroplasty allowed higher strength and a better recovery than lower frequencies [68,69]. Stimulation intensity was determined from the motor threshold (MT). Then, intensity was increased by 0.1 × MT at each session from 1.1× MT (during the first session) to 2× MT (during the last session).

### 4.7. Muscle and Bone Sampling

At the end of the NMES session, animals from all groups were euthanized with a lethal dose of anesthetic. FD and TA muscles were removed. Sampling was performed 90 min after the last stimulation in stimulated groups. The implanted hindpaw was incised from knee to ankle and FD and TA muscles were separated from the surrounding tissue and carefully collected after section of the proximal and distal tendons. Immediately after collection, muscles were frozen in isopentane (2-méthylbuthane, Sigma-Aldrich^®^) and stored at −80 °C for further immunohistochemistry analysis.

Implanted rat tibial bones were cut at both extremities (at least 4 mm on either side of the implant) using a micro-circular saw (Dremel 300 series multitool, Bosch^®^), removed, and stored at −20 °C before implant-to-bone adhesion measurement and analysis of the implant surface.

### 4.8. Implant-To-Bone Adhesion

Sectioned tibial bones were placed, implants downward, on a flat metal bracket containing a groove, allowing the implant to be loaded by a tension device (Figure 5B). A specific gripper was machined for this tensile test. The clamping of the gripper on the implant is ensured by a bolt. Highly resistant wire was passed through the gripper to apply the tensile load. The mobile part of the testing device was slowly displaced to stretch the wire until the implant loosened. The applied displacement was controlled by a numerical axis with controlled speed displacement (0.02 m/min). During this experiment, the value of the force was recorded using a 10 Hz frequency using a dynamometer sensor with 0.01 N of resolution (Kistler^®^, Les Ulis, France). The maximum load value was obtained just before the breakout of the implant. It was recorded by the acquisition software linked to the dynamometer sensor (Kistler^®^). This value reflected the maximum tensile force required to loosen the implant.

### 4.9. Muscle Cell Death Analysis

FD and TA muscles were cut into 50 μm-thick longitudinal slices using a cryostat (Leica Microsystems^®^, Wetzlar, Germany), collected on glass slides (Superfrost Plus Slat^®^, Thermo Scientific^®^, Waltham, MA, USA), rehydrated in a phosphate buffer solution (PBS), and then immersed in PBS containing 10% normal donkey serum (NDS) and 0.2% Triton X-100. Sections were then incubated overnight in the same blocking solution with a rabbit anti-activated caspase 3 (1/1000, Cell Signaling Technology^®^, Beverly, MA, USA). Following several washes in PBS, sections were incubated for 2h in PBS containing 5% NDS with Alexa Fluor^®^ 488 donkey anti-rabbit antibody (1/400, Invitrogen^®^, Carlsbad, CA, USA). Activated caspase 3 positive cells were quantified in muscles using an epifluorescence microscope (Leica Microsystems^®^) to determine the apoptosis rate as the average number of dead cells per area of 0.3 cm^2^.

### 4.10. Statistical Analysis

The statistical treatment was performed using the R Instat software (GraphPad Software, La Jolla, CA, USA) and data was expressed as mean ± standard error of the mean (SEM). Prior to any statistical test, normal distribution was checked with the D’Agostino-Pearson omnibus K2 test. According to this result, we selected the parametric or nonparametric statistical test. Thus, pull-out strength results were compared using the Kruskall–Wallis test followed by the Dunn post-hoc test. Analysis of variance (group effect) was performed to compare the muscle number of dead cells. Significant effects were considered when *p* < 0.05, and a post-hoc Newman–Keuls test was therefore applied.

## 5. Conclusions

This study assesses the effect of a high-salt diet, associated (or not) with two weeks of the NMES program currently used in human rehabilitation, on the bone adhesion of four common biomaterials (metallic: Ti6Al4V and CrCo and non-metallic: PEEK and Al_2_O_3_) and on the viability of muscle cells around the implant. The main result is that a high-salt diet is deleterious for muscle cell survival whatever the material implanted. Furthermore, except for the Ti6Al4V implant, the electrical current increases this deleterious effect on direct stimulated muscle tissue. Thus, the combination of a high-salt diet and rehabilitation with electrical stimulation for people undergoing arthroplasty should be avoided. The next experiments will have to determine the underlying mechanisms that lead to cell death in the presence of non-metallic alloys and in the condition of a high-salt diet.
